# Examining the relationship between genetic polymorphisms (BDKRB2, GNB3, HIF1A, MCT1, NOS3) and endurance athlete status

**DOI:** 10.1007/s00421-024-05498-z

**Published:** 2024-05-16

**Authors:** Gökhan İpekoğlu, Necdet Apaydın, Tuğba Çetin, Ahsen Nur Eren, Pelinsu Topçu, Büşra Yücelsoy, Güngör Civelek, Mert Sakar

**Affiliations:** 1 Faculty of Sport Science, Ordu University, Ordu, Turkey; 2 School of Physical Education and Sports, Karabuk University, Karabuk, Turkey

**Keywords:** BDKRB2, GNB3, HIF1A, MCT1, NOS3, Genetic polymorphism

## Abstract

**Purpose:**

Genetic factors are important in terms of athletic performance. Recent studies to determine the relationship between the genes that lead to physiological responses have attracted attention. In this respect, this meta-analysis study was designed to examine the relationship between genetic polymorphism (BDKRB2 rs5810761, GNB3 rs5443, HIF1A rs11549565, MCT1 rs1049434, NOS3 rs2070744) and endurance athlete’s status.

**Methods:**

The search included studies published from 2009 to 2022. To determine the relevant studies, Pubmed, Web of Science databases were systematically scanned. Only case-control studies were included in the meta-analysis. To determine the relevant studies, Pubmed, Web of Science databases were systematically scanned, and a total of 31 studies met the criteria for inclusion in the meta-analysis. Relevant data from the included studies were collected and analyzed using a random effects or fixed effects model. The effect size was calculated as the odds ratio or a risk ratio the corresponding 95% confidence intervals.

**Results:**

According to the results of the analysis, BDKRB2 rs5810761 + 9 allele, and NOS3 rs2070744 T allele were significantly more prevalent in endurance athletes (*p* < 0.05). Genotype distributions of BDKRB2 rs5810761, MCT1 rs1049434, and NOS3 rs2070744 showed significant differences in the dominant model (*p* < 0.05). However, no significant association was found between endurance athlete status and GNB3 rs5443 and HIF1A rs11549465 polymorphisms.

**Conclusion:**

These results show that some gene polymorphisms play an important role in endurance athlete status and suggest that having a specific genetic basis may also confer a physiological advantage for performance.

## Introduction

Athletic performance is a complex characteristic influenced by various factors such as training, gender, and social status (Varillas-Delgado et al. 2022). However, recent studies have shown the importance of genetic factors in determining both endurance and strength athlete status through the identification of genetic markers (Yang et al. 2003; Ruiz et al. 2010; Pimenta et al. 2013; Grenda et al. 2014; Sawczuk et al. 2015; Rankinen et al. 2016; Díaz Ramírez et al. 2020; Neto et al. 2022; Varillas-Delgado et al. 2022; Ipekoglu et al. 2022, 2023). The discovery of genetic markers has significantly influenced the field of sports science today, contributing to our understanding of the complex relationship between genetics and anaerobic performance. The identification of genetic markers helps us understand genetic variations among individuals and their effects on sports performance. For instance, it has been found that individuals with specific genetic characteristics are more likely to have a high strength or speed profile (Díaz Ramírez et al. 2020). This finding may provide a significant perspective by indicating a potential dominant inheritance model. On the other hand, endurance performance, in its most fundamental sense, requires the ability to deliver and utilize oxygen in working muscles (Guth and Roth 2013). As Joyner (2019) notes, VO_2max_ can be an important indicator that represents the upper limit of aerobic capacity and can be sustained for longer periods of time. Its physiological determinant is that red blood cells transport oxygen to the tissues with the help of hemoglobin and pump oxygenated blood to skeletal muscles at the same time (Lundby et al. 2017). In a study based on the correlation between differential gene expression and physiological performance parameters, it was found that five genes could explain 42% of VO_2peak_ variation (Reitzner et al. 2024). What is particularly intriguing is the ability of specific genes to account for a significant portion of an important performance metric such as VO_2peak_. This raises the importance of genes that may play a role in defining endurance performance, especially those related to metrics like VO_2max_. This should also be taken into account for achieving optimal performance. Specific genetic variants in the athlete's genotype are believed to be one of the elements that enable athletes to reach their highest possible performance levels. When examining the literature, the genes NOS3 (Nitric Oxide Synthase 3), MCT1 (Monocarboxylate transporter 1), BDKRB2 (Bradykinin receptor B2), GNB3 (Guanine nucleotide-binding protein), and HIF1 (Hypoxia-Inducible Factor-1A) have been frequently associated with endurance and energy metabolism ((Ahmetov and Fedotovskaya 2015; Semenova et al., 2023).

Bradykinin 2 receptor (BDKRB2) is encoded by the BDKRB2 gene, which is located on chromosome 14q32 and is expressed in most human tissues. The insertion/deletion polymorphism of 9 base pairs (-9/ + 9, rs5810761) in exon 1 is the most frequently studied polymorphism concerning the relationship between genotypes and athletic status (Williams et al. 2004). The -9bp allele is associated with high gene transcriptional activity, high mRNA expression, and increased receptor activity (Braun et al. 1996; Lung et al. 1997).

The GNB3 gene codes for the beta 3 subunit of guanine nucleotide-binding proteins. A polymorphism C825T in exon 10 of the GNB3 (rs5443) gene has been identified. These nucleotide-binding proteins play a significant role in defining the cellular response to stimuli by integrating signals between receptor and effector proteins, leading to physiological responses in tissues and organisms (Ahmetov and Fedotovskaya 2015). Furthermore, aerobic exercises with performance-enhancing properties have a significant effect on skeletal muscle metabolic pathways (Eynon et al. 2009). In a study by Faruque et al. (2009), a significant relationship between the T allele of GNB3 C825T polymorphism and peak oxygen consumption was observed.

HIF-1, a heterodimer consisting of the hypoxia-inducible factor-1 alpha (HIF-1a) and hypoxia-inducible factor-1 beta (HIF-1b), is one such genetic factor. The expression of HIF-1a and its protein level are dependent on blood oxygen levels and pressure (Ahmetov et al. 2008). The HIF-1 gene, which is involved in oxygen homeostasis, is responsible for the transcription of more than 60 proteins, in addition to erythropoietin and VEGF. These proteins increase oxygen levels by promoting erythropoiesis and angiogenesis, thereby activating genes involved in glucose transport processes (Semenza, 2003). Prior et al. (2003) stated that HIF-1a PRO/PRO homozygotes maintain their ability to increase VO_2max_ through aerobic exercise.

The MCT1 gene (monocarboxylate transporter 1), also known as SLC16A1, is a member of metabolic genes (Dzitkowska-Zabielska et al. 2022). The MCT1 gene encodes a cotransport that carries blood lactate to type I muscle fibers in oxidative muscle fibers (Saito et al. 2021). Green et al. (2002) found that a prolonged endurance exercise session (%60 maxVO_2_ for 5–6 h) could increase MCT1 protein expression and decrease lactate levels in the muscles due to high transport and lifting rates.

The NOS3 gene, located on human chromosome 7q35-36, encodes endothelial nitric oxide synthase, which converts L-arginine to L-citrulline and nitric oxide (NO) (Eynon et al. 2012). This gene is another candidate gene to explain human variations in health and exercise-related phenotype (Gómez-Gallego et al. 2009). Several recent studies have identified associations between human variations in health and exercise-related phenotypes and the NOS3 gene (Vimaleswaran et al. 2008).

The NOS3 gene, for instance, can enhance muscle blood flow by increasing nitric oxide production. This enhancement in blood flow is crucial for delivering oxygen and nutrients to active muscles, thus contributing to improved endurance performance; the MCT1 gene may influence endurance by regulating cellular energy metabolism; the BDKRB2 gene can enhance oxygen transport by supporting blood vessel dilation; the GNB3 gene may impact energy production by influencing proteins controlling energy metabolism; and the HIF1 gene can affect endurance by regulating cellular oxygen levels. Wouldn't examining the role of relevant alleles and genotypes based on the functions genetic mechanisms could have, lead us to more concrete information? Evaluating genetic information can contribute to athletes and coaches making informed decisions tailored to the unique characteristics of each individual, thereby facilitating the unlocking of their full potential (Weyerstraß et al. 2018). Identifying specific genetic markers can help understand an individual's predisposition to certain physical attributes or performance capacities. Particularly, investigating different genetic variants between case–control groups and analyzing the distribution of alleles and genotypes may contribute to a better understanding of the physiological effects of these genetic characteristics and their potential contributions to endurance performance. One of the initial studies that will be a crucial step in determining whether athletes and normal individuals show similarity or difference in genotype and allele frequency distributions is thought to be. This could be supported by a robust meta-analysis to provide a comprehensive perspective. Therefore, meta-analysis can help us understand the effects of specific genetic variants and genotypes on endurance performance more precisely by synthesizing comprehensive data obtained from various studies. This situation facilitates genetic counseling, enabling the creation of more personalized training and education programs for athletes based on their genetic profiles. Thus, the aim of this meta-analysis study is to investigate the distribution frequencies of five genetic polymorphisms (BDKRB2, GNB3, HIF1A, MCT1, NOS3) in endurance athletes and normal individuals, and to interpret the relationship of these genes with endurance athlete status.

## Materials and methods

### Search strategy

To identify relevant studies on BDKRB2, GNB3, HIF1A, MCT1, NOS3 gene polymorphisms and endurance athlete status, a comprehensive search of electronic databases including PubMed and Web of Science was conducted. The population to be examined within the scope of the research included endurance athletes for the case groups and sedentary individuals for the control participants. The database search included all studies conducted on the subject (from the first study in 2009 to the last study in 2022) and utilized the following keywords: “BDKRB2 polymorhism”, “GNB3 polymorhism”, “HIF1α polymorhism”, “MCT1 polymorhism”, “NOS3 polymorhism”, “genetic polymorphism”, in combination with “athlete” and “endurance”. The search was limited to studies published in English and included original research. The "Related articles" feature in the electronic databases was used to identify additional studies that may have been missed in the initial search. The reference lists of relevant studies were also searched to identify additional studies. Experts in the field were contacted and asked for any additional studies they were aware of that had not been identified in the search. During the database search using keywords, attention was initially given to the titles of the studies, and those studies with titles potentially relevant to the subject of meta-analysis were saved. Subsequently, the abstracts of the studies were reviewed, and studies that did not meet the inclusion criteria were excluded from the analysis. The studies that passed this filtering stage were then examined in full text, and those meeting the inclusion criteria were included in the meta-analysis.

### Inclusion and exclusion criteria

A total of 567 studies were initially identified, of which 31 met the inclusion criteria for the meta-analysis. Studies were included in the meta-analysis if they met the following inclusion criteria: (1) examined the association between the BDKRB2 (rs5810761), GNB3 (rs5443), HIF1A (rs11549465), MCT1 (rs1049434), NOS3 (rs2070744) gene polymorphisms and endurance athletes status; (2) the study included case and control group, (3) the study provided sufficient data to calculate an odds ratio or risk ratio with 95% confidence intervals, and (4) studies that are full text in English. The criteria for exclusion encompassed the following: (1) studies classified as review articles, (2) studies categorized as case reports, (3) studies that did not provide sufficient data to calculate an odds ratio or risk ratio and did not include 95% confidence intervals for these calculations, and (4) studies that are not full text in English.

### Data extraction

Two reviewers independently extracted data from the identified studies using a standardized data extraction form. The following data points were extracted from each study: (1) study characteristics (e.g. study design, sample size, population characteristics); (2) details on the gene polymorphisms (e.g. genotyping method, allele frequencies); (3) results of the association between the BDKRB2, GNB3, HIF1A, MCT1, NOS3 gene polymorphisms and endurance athlete status (e.g. odds ratios). Any discrepancies in data extraction were resolved through discussion and consensus between the reviewers.

### Eligibility criteria

Athlete-control articles including BDKRB2, GNB3, HIF1A, MCT1 and NOS3 gene polymorphisms associated with endurance sports were included in the study. On the other hand, meta-analyses that contained unclear data, did not include a control group, and those of which full text could not be available were excluded from this meta-analysis.

### Quality assessment

**Fig. 1:**
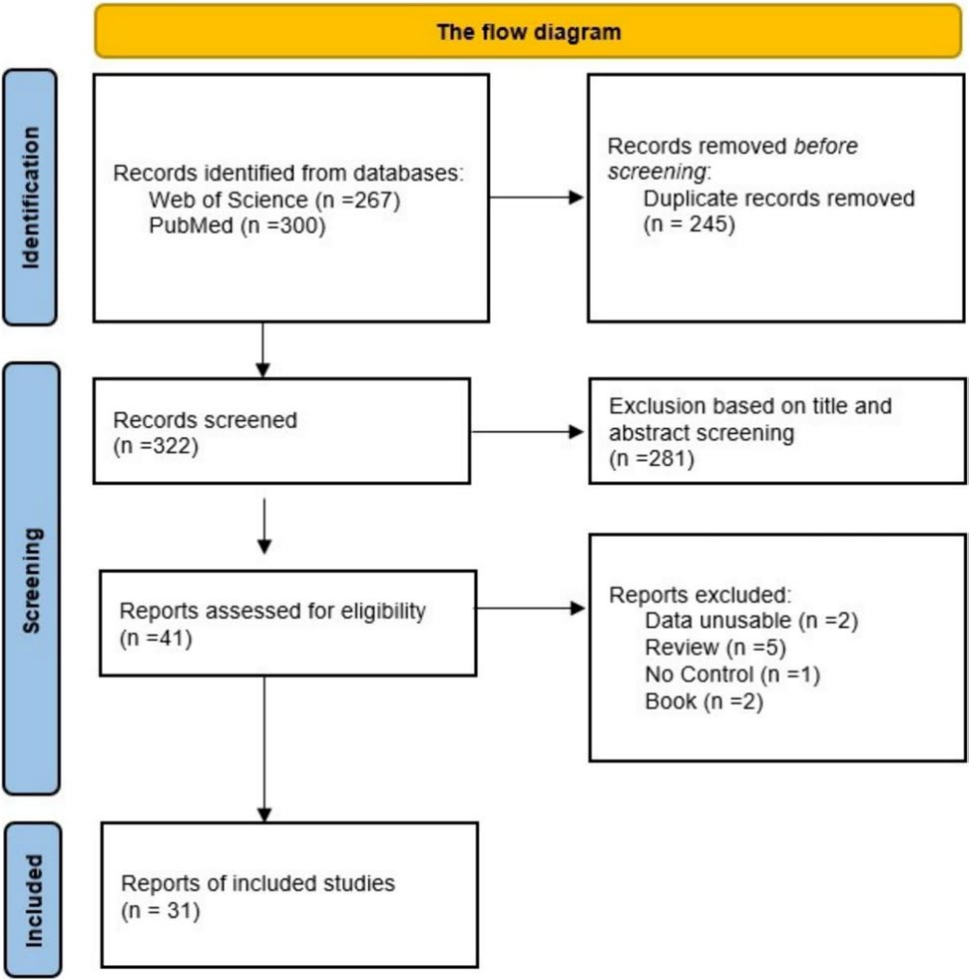
The flow diagram of included/excluded studies

The quality of the studies was assessed using the Newcastle–Ottawa Scale. The Newcastle–Ottawa Scale provides a structured framework for evaluating the quality of non-randomized studies and helps researchers make informed decisions regarding the inclusion of studies in systematic reviews and meta-analyses. According to the results of the Newcastle–Ottawa Scale, studies with higher scores are generally acknowledged to have lower risk of bias and higher methodological quality. The scale was applied by two different authors, and any discrepancies in interpretation between the authors were discussed and resolved. Each study is evaluated based on eight items of the NOS, which are divided into three groups: selection of study groups (four items), comparability of groups (one item), and ascertainment of outcome of interest for cohort studies (three items). Except for the 5th item for cohort studies, each item can be awarded a maximum of one star. However, if the study answers both questions in the 5th item, it can receive 2 stars. As a result of the authors' consensus, studies receiving 7 stars, or more are considered to meet adequate quality criteria (Fig. 1).

### Statistical analysis

The statistical analysis involved pooling the data from the included studies using either random effects or fixed effects models, owing to the substantial heterogeneity observed among the studies. The presence of heterogeneity was evaluated through the Cochran *Q* test and the *I*^2^(%) statistic (Semenova et al. 2020). Publication bias was assessed using funnel plots and the Egger test. The effect size was computed as the odds ratio or risk ratio, accompanied by 95% confidence intervals. When three or more studies were identified for the sub-dimension analysis, the relevant sub-dimension was included in the meta-analysis. In this study, utilizing a case–control design, we examined the polymorphism distributions between endurance athletes and healthy, physically active control individuals. Sub-dimensions such as the type of endurance sport, athlete status (elite athlete status), gender, and ethnicity were identified, and the polymorphism distributions were analyzed using statistical methods for comparison with those of the control group. The statistical analysis was conducted using Jamovi 2.3.

## Results

In this meta-analysis, a search was conducted using specific keywords in the PubMed and Web of Science databases, resulting in the identification of 567 articles. After reviewing titles, abstracts, and full texts, 31 articles were deemed suitable for inclusion in this meta-analysis. During the examination of the articles, factors such as the absence of a control group, the lack of a group consisting of endurance athletes, and unclear allele/genotype data were considered as limiting factors, and articles containing these factors were not included in the meta-analysis (Table 1).

**Table 1:** Descriptive information of the studies included in the meta-analysis

Study	Race	Name genes (rs number)	Total athletes	Total controls	Newcastle–Ottawa scale			
					Sel	Com	Exp	Total
Grenda et al. 2015	E	GNB3 (rs5443)	50	379	4	2	3	9
Bosnyak et al., 2020	C	HIF1A (rs115449465)	110	90	4	2	3	9
Cıeszczyk et al., 2012	E	HIF1A (rs115449465)	127	83	4	1	3	8
Döring et al. 2010	C	HIF1A (rs115449465)	303	295	3	1	3	7
Drozdovska et al. 2013	C	HIF1A (rs115449465)	81	260	4	1	3	8
Drozdovska et al. 2013	C	NOS3 (rs2070744)	82	321	4	1	3	8
Bosnyak et al., 2020	E	GNB3 (rs5443)	110	90	4	2	3	9
Eynon et al. 2010	C	HIF1A (rs115449465)	74	240	4	1	3	8
Eynon et al. 2009	C	GNB3 (rs5443)	74	234	4	2	3	9
Eynon et al. 2011	C	BDKRB2 (rs5810761)	74	240	4	2	3	9
Fedotovskaya et al. 2014	C	MCT1 (rs1049434)	142	467	4	1	3	8
Gómez-Gallego et al. 2009	C	NOS3 (rs2070744)	100	100	4	1	3	8
Gómez-Gallego et al. 2010	C	GNB3 (rs5443)	100	100	4	2	3	9
Grenda et al. 2014	E	BDKRB2 (rs5810761)	47	230	4	1	3	8
Guilherme et al. 2021	E, C	MCT1 (rs1049434)	633	1442	4	2	3	9
Neto et al. 2022	C	BDKRB2 (rs5810761)	26	1570	4	1	3	8
Ruiz et al., 2011	C	GNB3 (rs5443)	174	340	4	1	3	8
Ruiz et al., 2010	C	NOS3 (rs2070744)	100	100	4	1	3	8
Saito et al. 2021	C	MCT1 (rs1049434)	226	1028	4	1	3	8
Saunders et al. 2006	C	BDKRB (rs5810761)	422	202	4	1	3	8
Sawczuk et al. 2014	C	GNB3 (rs5443)	123	354	4	1	3	8
Sawczuk et al. 2015	C	MCT1 (rs1049434)	112	621	4	1	3	8
Sessa et al. 2011	E	NOS3 (rs2070744)	53	38	4	1	3	8
Varillas-Delgado et al. 2021	C	NOS3 (rs2070744), BDKRB2 (rs5810761)	123	122	4	1	3	8
Varillas-Delgado et al. 2022	C	NOS3 (rs2070744), BDKRB2 (rs5810761)	160	160	4	1	3	8
Piscina-Viúdez et al. 2021	C	MCT1 (rs1049434)	85	107	4	2	3	9
Yvert et al. 2016	A	GNB3 (rs5443)	174	649	4	1	3	8
Dzitkowska-Zabielska., 2022	C	MCT1 (rs1049434)	48	41	4	1	3	8
Ben Zaken et al., 2015	A	MCT1 (rs1049434)	146	128	4	1	3	8
Zmijewski et al. 2016	C	BDKRB2 (rs5810761)	100	230	4	1	3	8
Zmijewski et al. 2018	C	NOS3 (rs2070744)	50	379	4	2	3	9

*A* Asian, *C* Caucasion, *E* European, *Sel* Selection, *Com* Comparability, *Exp* Explosure

### The BDKRB2 (rs5810761) allele and genotype distirbution (endurance vs control)

**Fig. 2:**
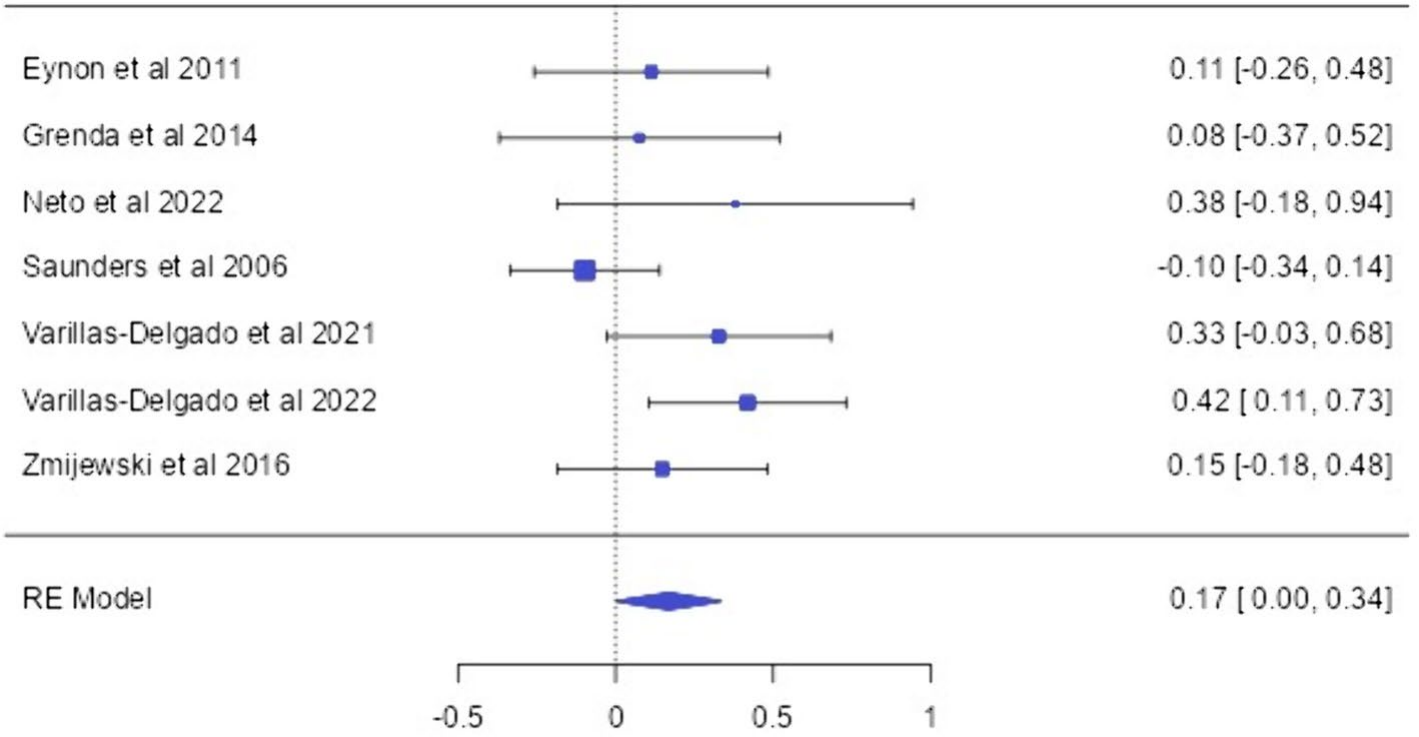
BDKRB2 -9/ + 9 allele-based OR (95%CI) and forrest plot

The relationship between the BDKRB (rs5810761) polymorphism and endurance athlete status has been included in seven articles (Eynon et al. 2011; Grenda et al. 2014; Neto et al. 2022; Saunders et al. 2006; Varillas-Delgado et al. 2021, 2022; Zmijewski et al. 2016). A total of 952 endurance athletes and 2754 controls were analyzed from these studies (Tables 2, 3 and 4). In subgroup analyses, at least 3 articles that met the criteria for gene-related conditions were considered. For the BDKRB2 (rs5810761) SNP, the frequency of the major allele (+ 9) was found to be higher in endurance athletes compared to the control group [OR: 0.17 [0.00; 0.34]] (*p* < 0.05). In the dominant model, endurance athletes were found to carry the wildtype + 9/ + 9 genotype distribution frequency more [OR: 0.33 [0.09; 0.57]] (*p* < 0.05). In subgroup analyses of the BDKRB2 (rs5810761) SNP, it was determined that male endurance athletes had a higher frequency of the major allele (+ 9) [OR: 0.17 [0.02; 0.33]] and + 9/ + 9 genotype distribution frequency [OR: 0.49 [0.16; 0.82]] compared to the control group (*p* < 0.05). When examining allele and genotype distributions in Caucasian endurance athletes, a significant difference was observed only in the dominant model [OR: 0.24 [0.04; 0.45]] (*p* < 0.05) (Fig. 2).

**Table 2:** Subgroup-analyses of the association between the allele-based OR of the investigated genes and endurance athlete status

	Meta analysis							Heterogeneity			Publ. Bias
Gene	Subgroup analysis	*n*	MA	OR	Lower CI	Upper CI	*p*	Heterogeneity *I*^2^	Heterogeneity Q (df)	*p* value of Q	Egger’s
BDKRB2 (rs5810761)	Overall	7	+ 9	0.17	0.00	0.34	**0.04**	36.58%	8.746	0.18	0.25
	Caucasian	6		0.08	− 0.05	0.22	0.23	7.01%	4.233	0.51	0.19
	Male	4		0.17	0.02	0.33	**0.03**	66.43%	8.938	0.03	0.01
	Swimmers	3		0.17	− 0.07	0.41	0.16	0%	0.721	0.69	0.59
GNB3 (rs5443)	Overall	7	C	− 0.06	− 0.24	0.11	0.47	45.89%	11.177	0.08	0.39
	Male	3		0.01	− 0.23;	0.26	0.90	0%	2.000	0.69	0.94
	Elite	3		0.13	− 0.12	0.38	0.31	24.83%	2.747	0.25	0.38
HIF1A (rs11549465)	Overall	5	Pro	− 0.01	− 0.23;	0.21	0.93	82.93%	23.426	0.00	0.02
	Rowers	3		− 0.45	− 0.78	− 0.13	**0.00**	72.77%	7.346	0.02	0.02
	Elite	4		0.01	− 0.26	0.28	0.93	86.03%	21.469	0.00	0.05
MCT1 (rs1049434)	Overall	7	T	0.08	− 0.01	0.18	0.80	76.28%	25.295	0.00	0.53
	Elite	4		0.02	− 0.10	0.13	0.75	82.94%	17.583	0.00	0.00
NOS3 (rs2070744)	Overall	7	T	0.21	0.06	0.36	**0.00**	85.21%	40.571	0.00	0.26
	Male	5		0.41	0.22	0.59	**0.00**	65.46%	11.579	0.02	0.45
	Elite	6		0.21	0.05	0.37	**0.00**	87.66%	40.533	0.00	0.03

Bold indicates the best result

*p < 0.05

*MA* major allele, *OR* odds ratio, *CI* confidence interval

**Table 3:** Subgroup-analyses of the association between the dominant model (DM) based OR of the investigated genes and endurance athlete status

Gene	Meta analysis							Heterogeneity			Publ. Bias
	Subgroup analysis	*n*	WT	OR	Lower CI	Upper CI	*p*	Heterogeneity *I*^2^	Heterogeneity Q (df)	*p* value of Q	Egger’s
BDKRB2 (rs5810761)	Overall	7	+ 9/ + 9	0.33	0.09	0.57	**0.00**	26.38%	7.463	0.28	0.83
	Caucasian	6		0.24	0.04	0.45	**0.02**	0%	2.930	0.71	0.99
	Male	4		0.49	0.16	0.82	**0.00**	40.07%	4.749	0.19	0.49
	Swimmers	3		0.13	− 0.24;	0.50	0.49	0%	0.318	0.85	0.74
GNB3 (rs5443)	Overall	7	C/C	− 0.06	− 0.28	0.17	0.61	36.83%	9.469	0.14	0.36
	Male	3		− 0.06	− 0.45	0.33	0.76	23.13%	2.437	0.29	0.88
	Elite	3		− 0.01	− 0.39	0.38	0.96	34.72%	3.085	0.21	0.51
HIF1A (rs11549465)	Overall	5	Pro/Pro	− 0.02	− 0.26	0.23	0.89	80.69%	20.715	0.00	0.01
	Rowers	3		− 0.47	− 0.83	− 0.12	**0.00**	68.15%	6.280	0.04	0.05
	Elite	4		0.05	− 0.25	0.35	0.73	83.08%	17.732	0.00	0.03
MCT1 (rs1049434)	Overall	7	T/T	0.17	0.02	0.32	**0.02**	0.01%	8.897	0.17	0.80
	Elite	4		0.23	− 0.01	0.47	0.05	16.35%	3.795	0.28	0.69
NOS3 (rs2070744)	Overall	7	T/T	0.29	0.08	0.51	**0.00**	81.78%	32.936	0.00	0.86
	Male	5		0.56	0.15	0.98	**0.00**	48.44%	7.253	0.12	0.94
	Elite	6		0.28	0.07	0.50	**0.01**	84.47%	32.188	0.00	0.00

Bold indicates the best result

*p < 0.05

*WA* wildtype, *OR* odds ratio, *CI* confidence interval

**Table 4:** Subgroup-analyses of the association between the recessive model (RM)-based OR of the investigated genes and endurance athlete status

	Meta analysis							Heterogeneity			Publ. Bias
Gene	Subgroup analysis	n	WT	OR	Lower CI	Upper CI	p	Heterogeneity *I*^2^	Heterogeneity Q (df)	*p* value of Q	Egger’s
BDKRB2 (rs5810761)	Overall	7	+ 9/ + 9	**− **0.09	**− **0.38	0.20	0.54	33.89%	8.422	0.20	0.02
	Caucasian	6		0.01	**− **0.26	0.28	0.93	29.67%	6.240	0.28	0.05
	Male	4		**− **0.03	**− **0.43	0.37	0.89	51.44%	6.254	0.10	0.02
	Swimmers	3		**− **0.34	**− **0.77	0.10	0.12	0%	0.814	0.66	0.50
GNB3 (rs5443)	Overall	7	C/C	0.14	**− **0.23	0.52	0.45	47.39%	11.320	0.07	0.64
	Male	3		**− **0.24	**− **0.82	0.33	0.40	0%	0.249	0.88	0.79
	Elite	3		− 0.57	1.08	− 0.06	**0.03**	0%	1.929	0.38	0.16
HIF1A (rs11549465)	Overall	5	Pro/Pro	**− **0.11	**− **1.39	1.17	0.87	40.43%	6.247	0.18	0.55
	Rowers	3		0.76	**− **0.62	2.14	0.28	13.3%	2.008	0.36	0.17
	Elite	4		0.40	**− **0.6	1.86	0.58	48.71%	5.810	0.12	0.78
MCT1 (rs1049434)	Overall	7	T/T	**− **0.04	**− **0.19	0.11	0.60	77.59%	26.775	0.00	0.35
	Elite	4		− 0.38	− 0.64	− 0.13	**0.00**	23.19%	23.19%	0.41	0.61
NOS3 (rs2070744)	Overall	7	T/T	**− **0.14	**− **0.41	0.13	0.31	65.34%	17.311	0.00	0.29
	Male	5		**− **0.29	**− **0.78	0.20	0.24	51.52%	8.833	0.07	0.29
	Elite	6		**− **0.14	**− **0.43	0.15	0.34	71.11%	17.309	0.00	0.28

Bold indicates the best result

*p <  0.05

*WA* wildtype, *OR* odds ratio, *CI* confidence interval

### The GNB3 (rs5443) allele and genotype distirbution (endurance vs control)

**Fig. 3:**
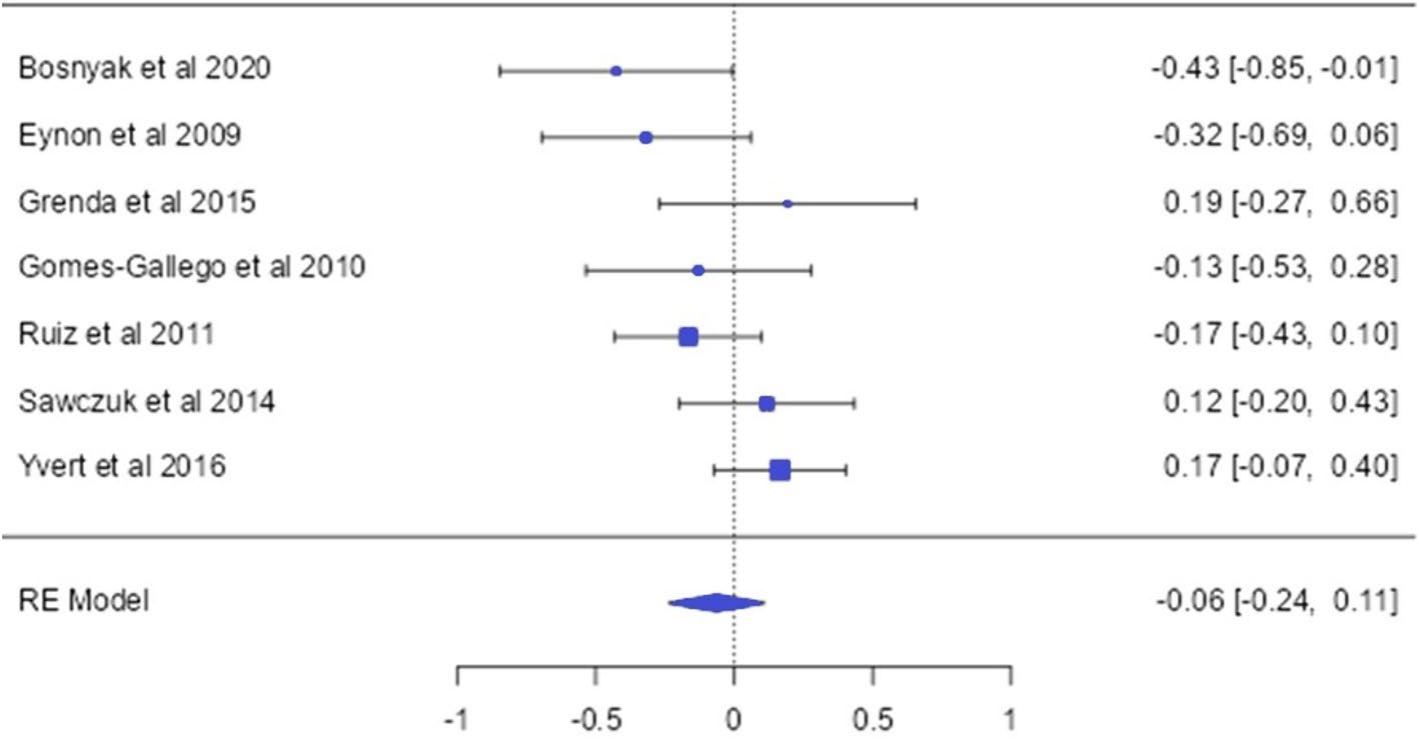
GNB3 C285T allele-based OR (95%CI) and forrest plot

A total of 805 endurance athletes and 2146 controls from a total of 7 studies related to the GNB3 gene polymorphism have been included in this study (Bosnyak et al. 2020; Eynon et al. 2009; Grenda et al. 2015; Gómez-Gallego et al. 2010; Ruiz et al. 2011; Sawczuk et al. 2014; Yvert et al. 2016). For the GNB3 (rs5443) SNP, the analysis revealed that the frequency of the recessive TT genotype in endurance athletes was lower [OR: 0.57 [− 1.08;− 0.06]] in the subgroup of elite athletes (*p* < 0.05) (Fig. 3).

### The HIF1A (rs11549465) allele and genotype distirbution (endurance vs control)

**Fig. 4:**
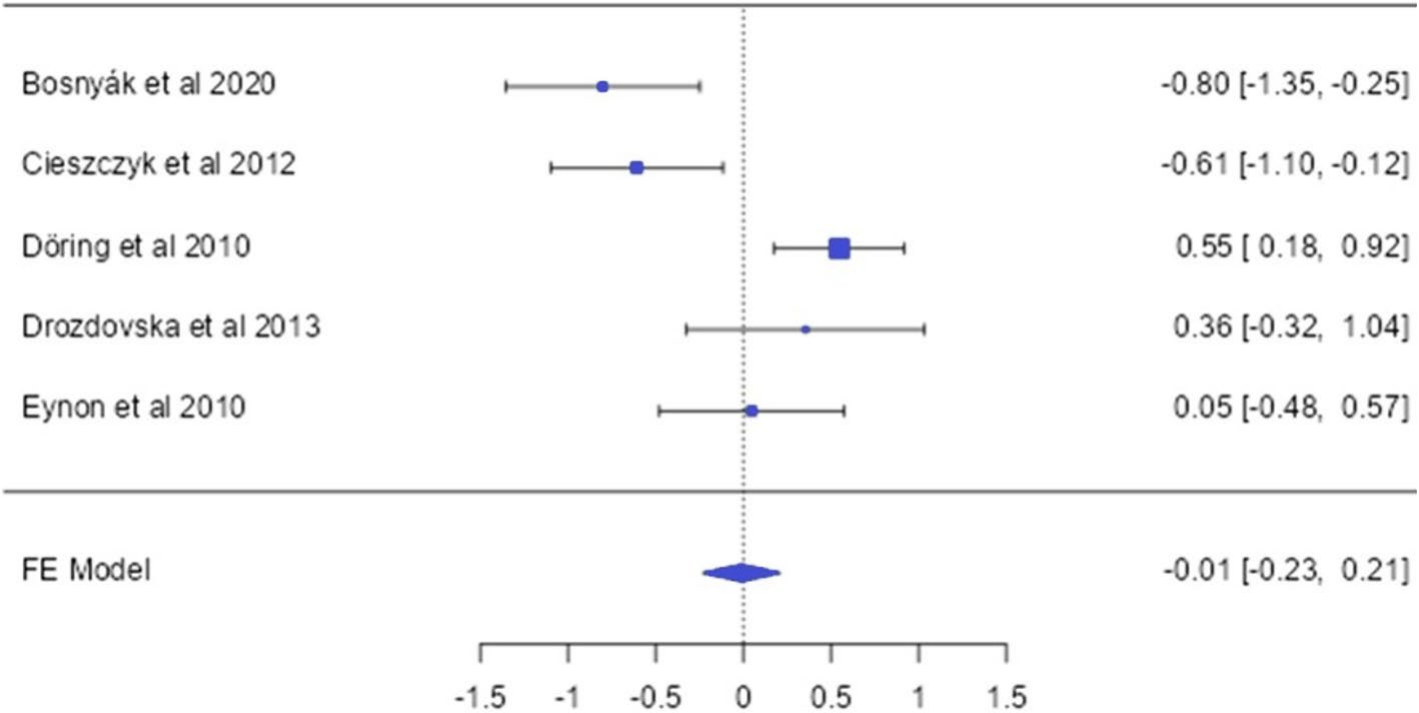
HIF1A Pro582Ser allele-based OR (95%CI) and forrest plot

From 5 studies investigating the distribution frequencies of HIF1A gene polymorphism in endurance athletes, 695 athletes and 968 controls were analyzed (Bosnyák et al. 2020; Cieszczyk et al. 2012; Döring et al. 2010; Drozdovska et al. 2013; Eynon et al. 2010). Regarding the HIF1A (rs11549465) SNP, significant differences were detected only in the rowers subgroup, with the major allele (Pro) [OR: − 0.45 [− 0.78; − 0.13]] and in the dominant model [OR: − 0.47 [− 0.83; − 0.12]] (*p* < 0.05) (Fig. 4).

### The MCT1 (rs1049434) allele and genotype distirbution (endurance vs control)

**Fig. 5:**
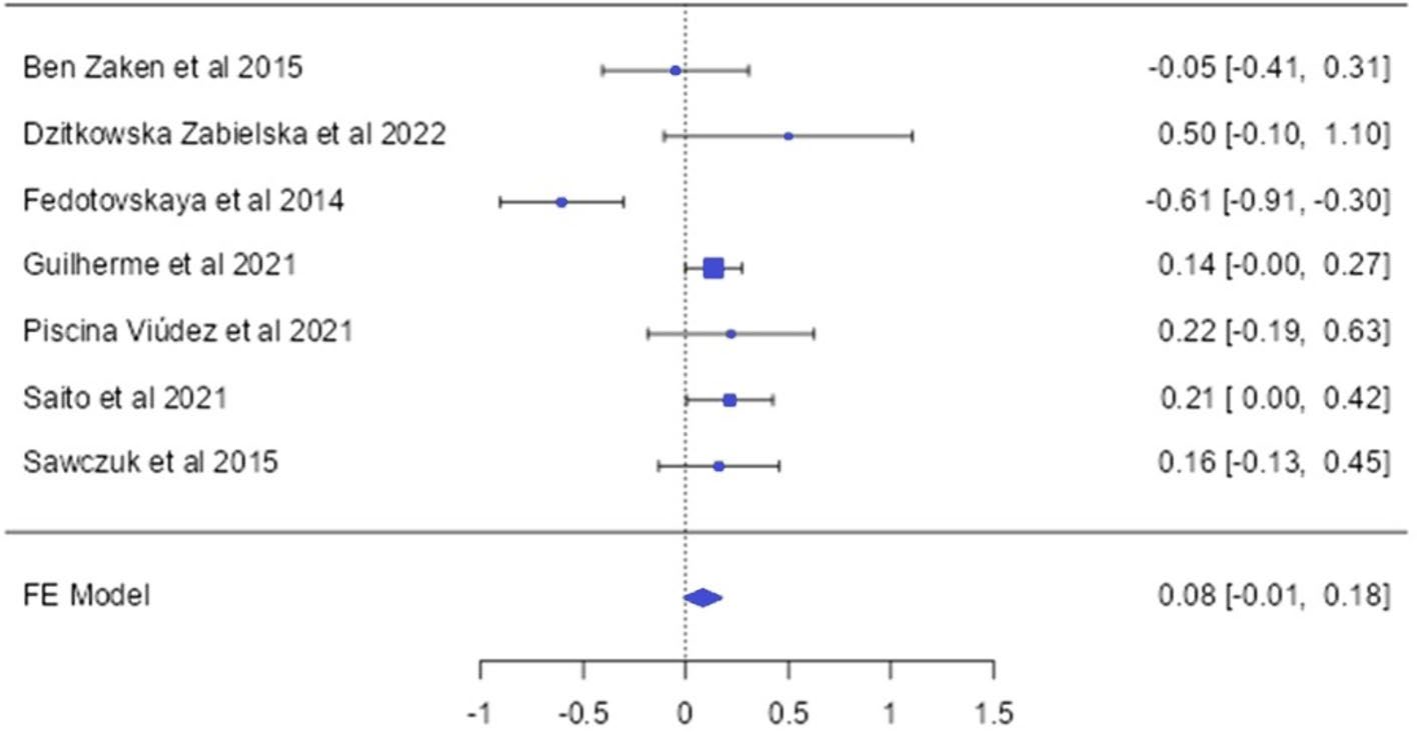
MCT1 A1470T allele-based OR (95%CI) and forrest plot

From 7 articles that met the inclusion criteria related to MCT1 gene polymorphism, 1392 endurance athletes and 3834 controls have been included in the study (Ben Zaken et al. 2015; Dzitkowska-Zabielska et al. 2022; Fedotovskaya et al. 2014; Guilherme et al. 2021; Piscina-Viúdez et al. 2021; Saito et al. 2021; Sawczuk et al. 2015). When analyzing the MCT1 (rs1049434) SNP genotypes, the overall analysis showed that endurance athletes [OR: 0.17 [0.02; 0.32]] and Caucasian athletes in the subgroup [OR: 0.42 [0.26; 0.58]] had a higher frequency of the T/T genotype (*p* < 0.05). In another subgroup, elite athletes had a lower frequency of the recessive AA genotype [OR: 0.38 [− 0.64;− 0.13]] compared to the control group (*p* < 0.05) (Fig. 5).

### The NOS3 (rs2070744) allele and genotype distirbution (endurance vs control)

**Fig. 6:**
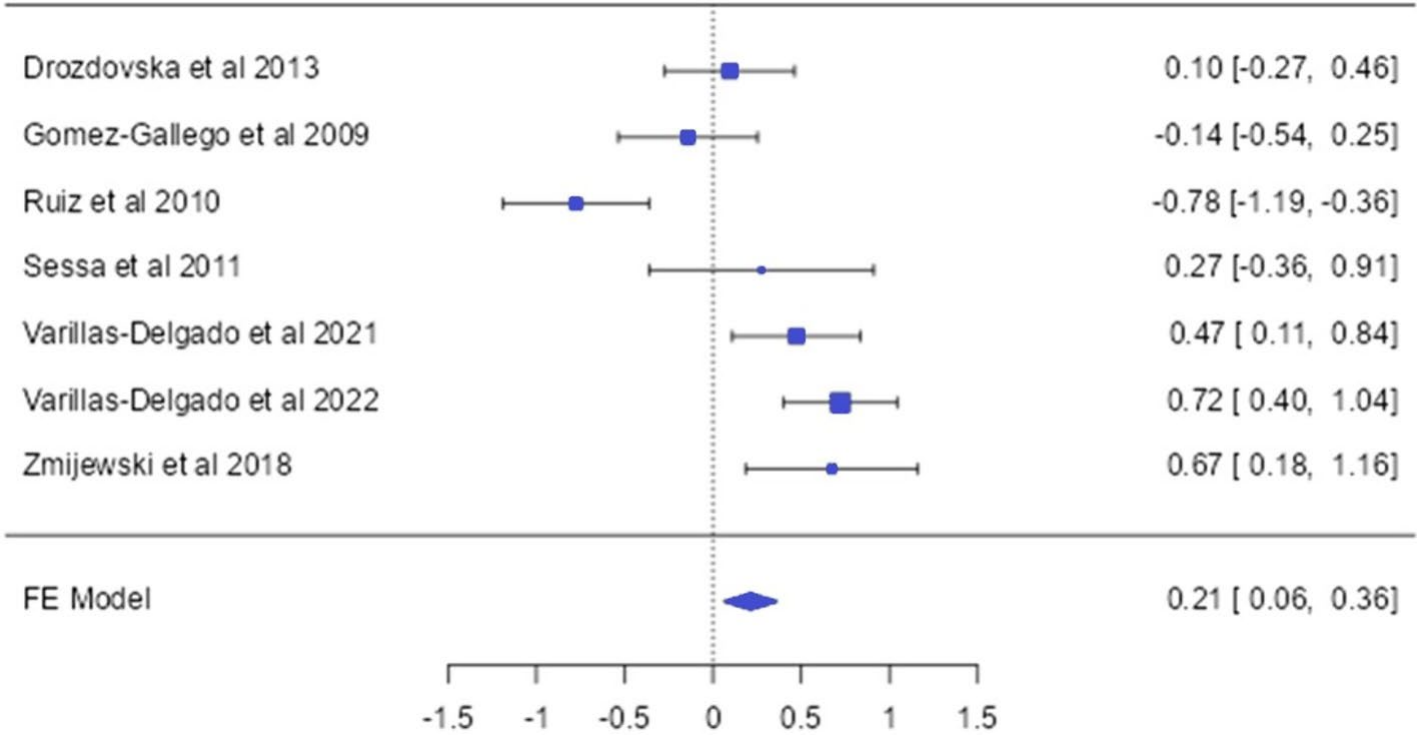
NOS3 -786 T/C allele-based OR (95%CI) and forrest plot

**Fig. 7:**
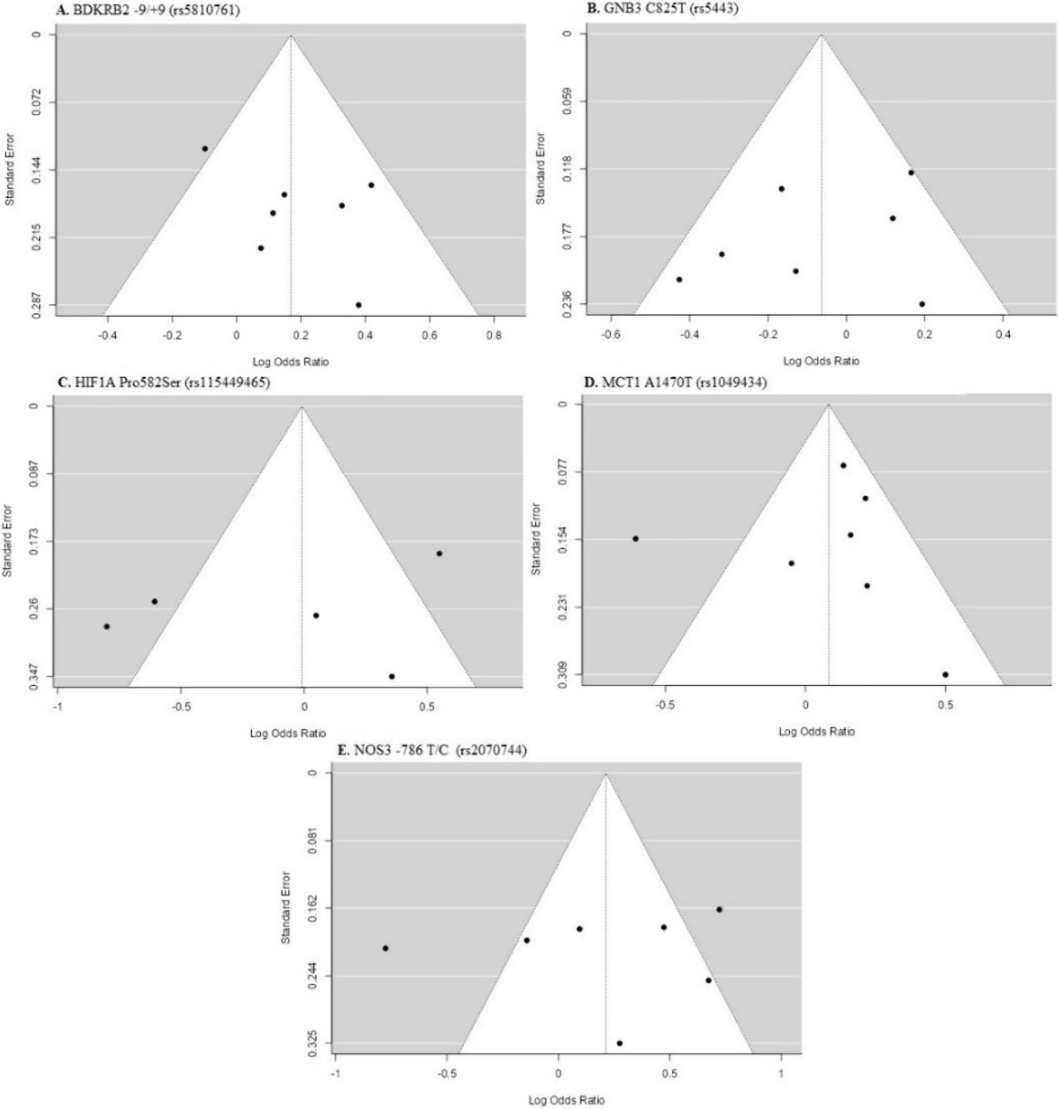
Allele based funnel plots for BDKRB2, GNB3, HIF1α, MCT1, NOS3 polymorphisms

From 7 studies examining the distribution of the NOS3 (rs2070744) SNP in endurance athletes, 668 endurance athletes and 1220 controls were included in the analysis (Drozdovska et al. 2013; Gómez-Gallego et al. 2009; Ruiz et al. 2010; Sessa et al. 2011; Varillas-Delgado et al. 2021, 2022; Zmijewski et al. 2018). Regarding the NOS3 (rs2070744) SNP, it was found that all articles included in the meta-analysis consisted of Caucasian endurance athletes. In the overall analysis of the NOS3 (rs2070744) polymorphism, significant differences were found in the frequency of the major allele (T) [OR: 0.21 [0.06; 0.36]] and in the dominant model [OR: 0.29 [0.08; 0.51]] in endurance athletes (*p* < 0.05). In subgroup analyses, male endurance athletes [OR: 0.41 [0.22; 0.59]] and elite endurance athletes [OR: 0.21 [0.05; 0.37]] had a higher frequency of the T allele compared to the control group (*p* < 0.05). Significant differences were also observed in the T/T genotype distributions for male endurance athletes [OR: 0.56 [0.15; 0.98]] and elite endurance athletes [OR: 0.28 [0.07; 0.11]] (*p* < 0.05). However, no significant differences were found in the recessive model in both overall and subgroup analyses (*p* > 0.05) (Figs. 6 and 7).

## Discussion

The aim of this study is to examine the allele and genotype distribution frequencies of five popular genes believed to be associated with endurance athlete status through a meta-analysis. A total of 31 articles published between 2009 and 2022 were found suitable for this meta-analysis. The polymorphism distributions of BDKRB2 (rs 5,810,761), GNB3 (rs 5443), HIF1A (rs 115,449,465), MCT1 (rs 1,049,434), and NOS3 (rs2070744) were evaluated and interpreted based on race, gender, sports discipline, and athlete level. All studies included in the meta-analysis underwent the Newcastle–Ottawa Scale assessment, and it was determined that they possessed sufficient methodological quality (≥ 7). When the studies included in the meta-analysis are examined in terms of effect size, it has been determined that the difference between groups in almost all polymorphisms can be explained by a small effect (Cohen’s *d* < 0.2). The origin of the small effect size is thought to be a general issue in studies related to genetic characteristics. When Egger's publication bias values were examined, it was observed that as the number of studies and participants decreased, especially in subdimensions, bias values increased (*p* < 0.05).

The BDKRB2 gene, considered an important vasodilator, facilitates high endurance performance by increasing glucose uptake in skeletal muscles and blood flow in the muscles during exercise (Grenda et al. 2014). In this study, the distribution frequencies of the BDKRB2 − 9/ + 9 (rs5810761) polymorphism were examined in a total of 3706 individuals, including 952 endurance athletes and 2754 controls. The analysis results showed that endurance athletes had a higher frequency of the BDKRB2 + 9 allele [OR = 0.17; 95% CI = 0.00; 0.34] and + 9/ + 9 genotype [OR = 0.33; 95% CI = 0.09; 0.57] compared to the control group. The studies exhibited low heterogeneity (*p* < 0.05; *I*^2^ = 36.58%). In addition, significant publication bias among the studies was not observed (*p* = 0.25 for Egger weighted regression analysis). When analyzed in subgroups, moderate heterogeneity (*p* < 0.05; *I*^2^ = 66.43%) was observed, and it was determined that the frequency of the dominant model was higher in Caucasian male athletes. The presence of publication bias was detected by the Egger test (*p* = 0.01), and it is believed that it may stem from methodological differences that could occur in some studies, as observed in the funnel plot graph. However, no statistically significant differences were observed in other subgroups. In a study by Zmijewski et al. (2016), it was found that swimmers with the + 9/ + 9 genotype showed greater improvement in swimming performance compared to those with the − 9/ + 9 genotype. Another study reported a higher prevalence of the BDKRB2 + 9 allele and + 9/ + 9 genotype in long-distance swimmers compared to the control group (Neto et al. 2022). The study by Williams et al. (2004) reported that activation of BDKRB2 leads to elevated glucose uptake in skeletal muscles during physical activity, increased muscle blood flow, and consequently, enhanced endurance performance. In contrast, a close relationship between the BDKRB2 − 9/− 9 genotype and Ironmen triathlete status was reported by Saunders et al. (2006). However, relevant studies have not shown an association between the BDKRB2 + 9/− 9 polymorphism and endurance athlete status (Eynon et al. 2011; Grenda et al. 2014; Varillas-Delgado et al. 2021, 2022). These results may stem from the limitations inherent in the studies. Differences in study populations, methodology, and statistical analyses among the studies could contribute to the inconsistency in findings. No other meta-analysis studies focusing on the relationship between the BDKRB2 + 9/− 9 polymorphism and endurance athlete status were found.

In this study, the relationship between the GNB3 C/T (rs5443) polymorphism's genotype and allele distribution frequencies and endurance athlete status were examined. The results demonstrated a consistent and homogeneous distribution frequency of the T/T genotype [OR = − 0.57; 95%CI = − 1.08;− 0.06] among elite athletes, suggesting a significant difference compared to the control group, with no indication of heterogeneity or publication bias across the studies. However, when other subgroups were analyzed, no significant relationship was observed between endurance athlete status and the GNB3 C/T polymorphism. Upon reviewing the literature, it was observed that Bosnyák et al. (2020) found a lower frequency of the C allele in endurance athletes compared to the control group. Similarly, Eynon et al. (2009) reported a higher frequency of the T/T genotype in endurance athletes compared to the control group. In contrast, Yvert et al. (2016) observed a higher frequency of the C allele in international endurance athletes. Nevertheless, studies consistent with our findings, which showed no statistically significant difference between endurance athlete status and this gene, have also been reported (Gómez-Gallego et al. 2010; Grenda et al. 2015; Ruiz et al. 2011; Sawczuk et al. 2014). The literature presents contradictory results regarding the relationship between the GNB3 C/T polymorphism and endurance athlete status. This inconsistency may be attributed to different sports disciplines, diverse populations, and insufficient sample sizes. Therefore, it is believed that this meta-analysis study could provide researchers with a broader perspective on the relationship between the GNB3 gene and endurance athlete status.

The transcription factor hypoxia-inducible factor 1 (HIF1) is an important regulator of oxygen homeostasis, induced during hypoxic conditions. Oxygen consumption and metabolism play a significant role in competitive athletic performance (Yan and Bai 2023). Especially in endurance athletes, where cardiorespiratory fitness is crucial, the HIF1A Pro582 variant has been observed to be higher (Döring et al. 2010). In a study conducted by CieSzczyk et al. (2012) with rowers, the HIF1A Pro/Ser genotype and Ser582 allele distributions were found to be higher compared to the control group. According to the results of this study, the frequency of the Pro allele [OR = − 0.45; 95% CI = − 0.78;− 0.13]] and Pro/Pro genotype [OR = − 0.47; 95% CI = − 0.83; − 0.12]] was lower in rowers than in the control group. However, it is important to note the presence of high heterogeneity (*p* < 0.05; *I*^2^ = 72.77%) and publication bias (*p* = 0.02) in similar analyses, which may impact the generalizability and reliability of the findings. However, no difference was observed in other subgroups when the HIF1A Pro582Ser polymorphism distribution frequencies were examined. The study by Bosnyák et al. (2020) supports our findings. When the results of this study are examined, although no relationship was found between the HIF1A Pro582Ser variant and athletic endurance status, other studies have suggested that this variant may be associated with strength athlete status (CieSzczyk et al. 2011; Drozdovska et al. 2013; Gabbasov et al. 2013). In fact, when the relevant studies are examined, it should also be noted that the primary function of the HIF1A Pro582Ser variant may be associated with its occurrence in conditions of oxygen deficiency. Although hereditary physiological parameters such as maxVO_2_ represent significant genetic components in the context of endurance performance (Moir et al. 2019), it should be considered under which conditions this situation is supported. Especially under hypoxic conditions, where muscle cells demand more energy due to increased oxygen requirements, it can be speculated that the HIF1A Pro582Ser variant may be associated with increased hypoxic resistance in muscle cells (Tanimoto et al. 2003). Under hypoxic conditions, acute sprint exercise conducted with eight endurance athletes has been shown to stimulate genes associated with glycolytic metabolism, mitochondrial function, and oxygen transport (Nava et al. 2022). These results may provide a perspective on the lack of association between the HIF1A Pro582Ser polymorphism and endurance athlete status.

This study examined the genotype and allele distribution frequencies of the MCT1 A1470T polymorphism in a total of 5226 individuals, including 1392 endurance athletes and 3834 controls. According to the analysis results, the frequency of the MCT1 T/T genotype was found to be higher in endurance athletes compared to the control group [OR = 0.17; 95% CI = 0.02; 0.32]. Furthermore, it is noteworthy that the analysis indicated minimal heterogeneity (*p* < 0.05; *I*^2^ = 0.01%) across studies and no evidence of publication bias (*p* = 0.80), enhancing the reliability and robustness of the findings. When analyzed in subgroups, the frequency of the T/T genotype was also higher in endurance athletes of Caucasian origin compared to the control group [OR = 0.42; 95 % CI = 0.26; 0.58]. Similar findings have been reported in other studies, where the MCT1 major (T) allele was associated with endurance athlete status and athletic performance (Guilherme et al. 2021; Saito et al. 2021). In a study by Piscina-Viúdez et al. (2021), although no statistically significant association was found between athletic performance and the MCT1 gene, the MCT T/T genotype was observed to be higher in triathletes compared to the control group. In another study comparing swimmers and runners, it was reported that the frequency of the T allele was higher in swimmers than in runners (Ben-Zaken et al. 2015). As is known, in sports disciplines requiring endurance, the predominant ATP requirement occurs through aerobic pathways. Particularly, under conditions where sufficient oxygen is provided, resulting in the formation of lactate contributing to ATP demand, hydrogen atoms in NAD and FAD carrier proteins (NADH and FADH) culminate in prolonged high-energy outcomes through chemical reactions within the mitochondria. On the contrary, the conversion of hydrogen atoms given to pyruvate into lactic acid can hinder chemical reactions, leading to the formation of lactate. Considering that the MCT1 gene encodes a cotransport that transports blood lactate to type I muscle fibers, it should be considered that lactate levels in muscles may decrease, allowing for prolonged continuation of movement. Endurance requires the ability to sustain intensity in exercise for an extended period, and therefore, the MCT1 A1470T polymorphism is believed to confer an advantage in endurance performance.

Nitric oxide plays a significant role in myocardial repair and regeneration, as well as in regulating blood flow and vascular tone (Otani 2009). The NOS3-786 T/C polymorphism is believed to have an impact on the provision of blood flow for endurance performance (Gómez-Gallego et al. 2009). However, upon reviewing the literature, no meta-analysis studies specifically focusing on the association between the NOS3-786 T/C polymorphism and endurance athlete status were found. In this study, the distribution frequencies of the NOS3-786 T/C (rs2070744) polymorphism were examined in a total of 1888 individuals, including 668 endurance athletes and 1220 controls. The analysis results indicated that the T allele [OR = 0.21; 95% CI = 0.06;0.36] and T/T genotype [OR = 0.29; 95% CI = 0.08;0.51] of the NOS3-786 T/C polymorphism were more prevalent in endurance athletes. Moreover, when analyzed in subgroups, the dominant model was observed to be more prevalent in Caucasian, elite, and male endurance athletes. However, it is important to note that there was high heterogeneity observed among studies, particularly in the elite subgroup(*p* < 0.05; *I*^2^ = 84.47%), which suggests significant variability in the findings across different studies. This high heterogeneity may be attributed to factors such as the varying sample sizes across studies, the diversity of sports disciplines among athletes, and the distinction between elite and non-elite athletes. Further exploration and subgroup analysis considering these factors are warranted to better understand the observed variability in the results. For example, while Gómez-Gallego et al. (2009) included a total of 100 endurance athletes, with 50 professional road cyclists in their study, Sessa et al. (2011) included a total of 53 endurance athletes consisting of football, basketball, and hockey players. The differences in sample composition among these studies are believed to influence the results of this meta-analysis and contribute to the observed high heterogeneity. These findings were consistent with the study conducted by (Varillas-Delgado et al. 2021), which also showed a higher frequency of the T/T genotype in endurance athletes compared to the control group. This similarity further supports our study's findings (Zmijewski et al. 2018). In another study with Caucasian athletes, the NOS3 T allele was reported to be associated with strength athlete status (Drozdovska et al. 2013). Based on these findings, it is suggested that carriers of the NOS3 T allele and T/T genotype may have an increased likelihood of having an endurance athlete status. The role of nitric oxide (NO) in the regulation of basal vascular tone and blood pressure is an important factor (Ciȩszczyk et al. 2010; Sessa et al. 2011). Considering this information, the NOS3 gene for elite endurance athlete performance can be considered as an important genetic marker.

## Limitations

Considering the limitations of the study, there are constraints such as the limited number of studies including female athletes or uncertainties regarding polymorphism distributions, despite aiming to include both male and female athletes. These limitations hinder a more detailed interpretation regarding the inclusivity of the study. Additionally, this study is restricted to access only the Medline and Web of Science databases. Although this limitation aims to increase the reliability of the study, the lack of utilization of other medical databases and resources may lead to an incomplete coverage of studies related to the subject matter. The scientific value of results obtained in genetic studies is also associated with the number of individuals included in the study. While it is observed that this database limitation has reduced the number of studies included in the research to a certain extent, it is still believed to enhance the quality of the study. Another limitation of the study is the issue of insufficient data for calculating Odds Ratios (ORs).

## Conclusions

The results of the meta-analysis have determined that certain genetic polymorphisms (SNPs) may be associated with endurance athlete status. Specifically, the BDKRB2 rs5810761 + 9 allel, and NOS3 rs2070744 T allele were found to be more prevalent in endurance athletes. When genotype distribution results were examined, BDKRB2 rs5810761 + 9 + 9, MCT1 rs1049434 T/T and NOS3 rs2070744 T/T polymorphism distributions were more common in endurance athletes. However, in our study, no significant relationship was found between the GNB3 (rs5443) and HIF1A (rs11549465) polymorphisms and endurance athlete status. These findings may contribute to the field of sports genetics by providing a concrete approach to understanding the relationship between genetic polymorphism and athlete status. Furthermore, considering these results emphasizes the importance of targeted interventions in optimizing sports performance. In conclusion, this study allows for a better management of strategies related to sports performance by highlighting the importance of considering genetic factors to maximize athletes' potential.

## Data Availability

The datasets used and analyzed during the current study are available from the corresponding author on reasonable request.

## Abbreviations

BDKRB2: Bradykinin 2 receptor
GNB3: Guanine nucleotide-binding proteins
HIF1A: Hypoxia-inducible factor-1 alpha
MCT1: Monocarboxylate transporter 1
NOS3: Nitric oxide synthase
MA: Major allele
OR: Odds ratio
CI: Confidence interval
R: Random effect
F: Fix effect
